# Cortical Pathology in Vanishing White Matter

**DOI:** 10.3390/cells11223581

**Published:** 2022-11-12

**Authors:** Jodie H. K. Man, Charlotte A. G. H. van Gelder, Marjolein Breur, Daniel Okkes, Douwe Molenaar, Sophie van der Sluis, Truus Abbink, Maarten Altelaar, Marjo S. van der Knaap, Marianna Bugiani

**Affiliations:** 1 Department of Child Neurology, Emma Children’s Hospital, Amsterdam University Medical Centers, VU University Amsterdam, 1081 HV Amsterdam, The Netherlands; 2 Amsterdam Leukodystrophy Center, Emma Children’s Hospital, Amsterdam University Medical Centers, 1081 HV Amsterdam, The Netherlands; 3 Molecular and Cellular Mechanisms, Amsterdam Neuroscience, 1081 HV Amsterdam, The Netherlands; 4 Biomolecular Mass Spectrometry and Proteomics, Bijvoet Center for Biomolecular Research and Utrecht Institute for Pharmaceutical Sciences, University of Utrecht, 3584 CS Utrecht, The Netherlands; 5 Netherlands Proteomics Center, 3584 CS Utrecht, The Netherlands; 6 Department of Systems Bioinformatics, VU University Amsterdam, 1081 HV Amsterdam, The Netherlands; 7 Department of Child and Adolescent Psychology and Psychiatry, Complex Trait Genetics, Amsterdam Neuroscience, VU University Medical Center, 1081 HV Amsterdam, The Netherlands; 8 Department of Integrative Neurophysiology, Center for Neurogenomics and Cognitive Research, VU University Amsterdam, 1081 HV Amsterdam, The Netherlands; 9 Department of Pathology, Amsterdam University Medical Centers, 1081 HV Amsterdam, The Netherlands

**Keywords:** leukodystrophy, astrocytopathy, vanishing white matter, cortex, proteomics, astrocytes

## Abstract

Vanishing white matter (VWM) is classified as a leukodystrophy with astrocytes as primary drivers in its pathogenesis. Magnetic resonance imaging has documented the progressive thinning of cortices in long-surviving patients. Routine histopathological analyses, however, have not yet pointed to cortical involvement in VWM. Here, we provide a comprehensive analysis of the VWM cortex. We employed high-resolution-mass-spectrometry-based proteomics and immunohistochemistry to gain insight into possible molecular disease mechanisms in the cortices of VWM patients. The proteome analysis revealed 268 differentially expressed proteins in the VWM cortices compared to the controls. A majority of these proteins formed a major protein interaction network. A subsequent gene ontology analysis identified enrichment for terms such as cellular metabolism, particularly mitochondrial activity. Importantly, some of the proteins with the most prominent changes in expression were found in astrocytes, indicating cortical astrocytic involvement. Indeed, we confirmed that VWM cortical astrocytes exhibit morphological changes and are less complex in structure than control cells. Our findings also suggest that these astrocytes are immature and not reactive. Taken together, we provide insights into cortical involvement in VWM, which has to be taken into account when developing therapeutic strategies.

## 1. Introduction

Vanishing white matter (VWM) is one of the more prevalent leukodystrophies [1]. The disease most often has its onset in young children, but may occur at any age from the prenatal period to senescence [2,3]. Patients experience chronic neurological decline and are susceptible to stressors, including fever, infections and minor trauma [3,4,5]. These stressors may lead to episodes of rapid deterioration, which may result in a coma and sometimes death, but are most often followed by a partial recovery [3,4,5]. VWM is caused by bi-allelic mutations in any of the 5 genes encoding the subunits of the eukaryotic translation initiation factor 2B (eIF2B) [6,7]. eIF2B is a housekeeping factor crucial for the translation initiation of mRNA into proteins [8]. It also orchestrates the integrated stress response (ISR), a cell protective mechanism activated in response to cellular stress [9]. eIF2B in this way serves as a central regulator of the mRNA translation rate under normal and stress conditions [6,7]. The ISR is constitutively deregulated in VWM [10].

In VWM, the brain white matter is allegedly selectively affected, showing a lack of myelin and cystic degeneration [3,4,5]. There is meagre reactive gliosis and no glial scarring [3,4,5,11]. In the affected areas, astrocytes are immature and dysmorphic, and they overexpress the delta subunit of the glial fibrillary acidic protein (GFAPδ), indicating an abnormal intermediate filament composition [11]. Additionally, oligodendrocyte maturation is halted in a premyelinating state, explaining the lack of myelin [3,4,5,11]. Axons are lost in cavitated areas or appear thinned and swollen in more preserved tissue [12,13,14]. Recent evidence has pointed to astrocytes as primary drivers in the pathogenesis of VWM, with secondary defects in oligodendrocytes and axons [14,15,16]. Co-cultures of oligodendrocyte progenitor cells (OPCs) and astrocytes derived from wild-type and VWM mice showed that VWM astrocytes halt wild-type OPC maturation, whereas mutant OPCs mature normally when cultured with wild-type astrocytes [15]. Additionally, VWM astrocytes induce axonal pathology in myelinating culture systems containing wild-type mouse neurons [14]. On the basis of these findings, VWM is categorized under the astrocytopathies [5].

Magnetic resonance imaging (MRI) documents the progressive thinning of the cortices in long-surviving patients [3]. Post-mortem routine analyses, however, have not pointed to any cortical pathology so far [4,17,18]. This finding prompted us to explore the cortex in VWM in more depth using high-resolution-mass-spectrometry-based proteomics.

## 2. Materials and Methods

### 2.1. Patients

Post-mortem brain tissue from 4 genetically proven VWM patients was collected at the Amsterdam UMC location, VU university Amsterdam (Amsterdam, The Netherlands). All patients had a childhood onset of VWM. The controls included 4 subjects without confounding structural and neuropathological abnormalities obtained from the Netherlands Brain Bank (NBB, www.brainbank.nl). Cause of death of the latter was cardio-respiratory arrest, possibly due to arrhythmia. Demographic features of controls and patients are shown in Table 1. Tissue was collected within 6 h post-mortem. This study focused on the cortex of the middle frontal gyrus. Informed consent was obtained in all cases. The study was approved by the institutional review board of the Amsterdam UMC location, VU University Amsterdam (Amsterdam, The Netherlands) and conducted according to the declaration of Helsinki.

### 2.2. Fast Immunohistochemistry and Laser Capture Microdissection

Twenty µm thick frozen tissue sections were mounted on glass slides coated with polyethylene naphthalate, fixed in 100% ethanol for 20 min, and subsequently air-dried and rehydrated twice in sterile H_2_O for 1 min. Next, tissue sections were incubated in toluidine (1% *w*/*v* in sterile H_2_O) for 1 min at room temperature and washed in sterile H_2_O 3 times for 1 min. Sections were then dehydrated in 100% ethanol twice for 3 min and air-dried. Laser capture microdissection was performed using a Leica LMD6500 system (Leica Microsystems, Wetzlar, Germany). All cortical layers from the middle frontal gyrus were included. Microdissected tissue samples of 100 mm^3^ were collected into adhesive caps (Zeiss, Göttingen, Germany) and stored at −80 °C until use.

### 2.3. In-Solution Protein Digestion

Microdissected samples were lysed, reduced and alkylated in lysis buffer (6 M guanidine hydrochloride, 5 mM tris(2-carboxyethyl)phosphine, 10 mM chloroacetamide and 100 mM Tris-HCl pH 8.0) in 50 mM ammonium bicarbonate with pH 8.0–8.5 for 10 min at 99 °C with mixing at 750 RPM. Samples were sonicated with 20 mg protein extraction beads (Diagenode, Seraing, Belgium) using a Bioruptor Plus (Diagenode, Seraing, Belgium) for 20 cycles with on/off pulses of 30 s. Protein (20 µg/sample) was then digested with Lys-C at an enzyme-to-substrate ratio of 1:100 at 37 °C for 4 h. Samples were next diluted to a final concentration of 2 M guanidine hydrochloride using 25 mM Tris-HCl (pH 8.0) and processed for overnight digestion with trypsin at an enzyme-to-substrate ratio of 1:100 at 37 °C. Peptide samples were then acidified using 10% formic acid with pH < 2.0. Finally, peptides were extracted using C18 cartridges (Agilent Technologies, Amstelveen, The Netherlands) and an automated AssayMAP Bravo Platform (Agilent Technologies, Amstelveen, The Netherlands) according to standard procedures. Eluted peptides were dried using a SpeedVac centrifuge at 37 °C and stored at −20 °C until analysis.

### 2.4. Mass Spectrometry Analysis

Peptide samples (1.5 µg/sample) were resuspended in 2% formic acid and analyzed using an Orbitrap Q-Exactive HF-X mass spectrometer (Thermo Fisher Scientific, Bleiswijk, The Netherlands) coupled to an UltiMate 3000 RSLCnano System (Thermo Fisher Scientific, Bleiswijk, The Netherlands). Peptides were loaded on a trap column (300 µm id × 5 mm, 5 µm particle size, reversed phase C18, Thermo Fisher Scientific, The Netherlands) at a flow rate of 10 µL/min in 100% solvent A (0.1% formic acid). Next, peptides were separated with an analytical column made in-house with solvent B (80% acetonitrile/0.1% formic acid) at a flow rate of 300 nL/min. The following gradient was used: 0–2.5 min with 92% solvent A; 2.5–157.5 min with 8% solvent B; 157.5–161 min with 35% solvent B; 161–165 min with 100% solvent B; and 165–175 min with 92% solvent A. Total run per sample was 175 min. Spectra were collected using a data-dependent acquisition method in which the top 15 precursors were selected from a full MS1 scan (*m*/*z* range = 315–1500, resolution = 60,000, target = 3 × 10^6^ ions) for MS2 analysis with higher-collision dissociation (target ions = 1 × 10^5^, max ion fill time = 50 milliseconds, isolation window = 1.4 *m*/*z*, normalized collision energy = 27%, resolution = 30,000). Precursor ions were excluded from fragmentation if charge state was unassigned or equal to 1, 6, 7, 8 or higher than 8. A dynamic exclusion of 16 s was included.

### 2.5. Mass Spectra Processing

RAW files were processed and searched against the Swiss-Prot human reference proteome database (20,431 entries, accessed on August 2019) using MaxQuant (version 1.6.6.0, Martinsried, Germany) [19]. Label-free quantification (LFQ) was performed by MaxLFQ with “match between runs” enabled, and minimal ratio count set at 2. False discovery rate (FDR) threshold was set to 1%. The minimum and maximum peptide length ranged from 7 to 25 amino acids. Carbamidomethylation of cysteine was set as fixed modification, and peptide N-terminal acetylation and methionine oxidation were set as variable modifications. A maximum of 2 missed trypsin cleavages was allowed. The maximum peptide and fragmentation mass tolerance was set at 20 ppm and 0.5 Da, respectively.

### 2.6. Differential Protein Expression Analysis

Statistical data analysis was conducted using R statistical software (version 1.3.959). Proteins identified as contaminants, only by site modification or in decoy reverse database were removed prior to analysis. Data were then log 2-transformed. Missing values were imputed based on a normal distribution in which the lowest detected LFQ intensity was set as mean and the average standard deviation of all detected proteins was set as standard deviation. Only proteins in which expression was detected in all subjects in either control or disease state were imputed. Differential protein expression analysis was carried out using the limma R package [20]. Statistical significance was set as *p*-value *<* 0.05 with Benjamini–Hochberg’s (BH) test for multiple comparison adjustment. Data were then further filtered to only include proteins showing at least ±1.5-fold change (FC). Hierarchical clustering analysis was performed using the ComplexHeatmap R package [21]. LFQ intensities were z-scored and clustered using the Euclidean and average methods as distance and clustering measures, respectively. The transcriptome dataset from human cortical fetal astrocytes, mature astrocytes, neurons, oligodendrocytes, and microglia (GSE73721) [22] was used to generate cell type marker lists. These lists were then used to identify potential cell-type-enriched protein expression changes in the proteome dataset. In this context, the term enriched refers to the expression of a marker in a specific cell type relative to its expression across all other cell types. Only markers enriched >5-fold in a given cell type over all other cell types were included. A Fisher’s exact test was performed to find significant cell type enrichment (*p*-value < 0.05).

### 2.7. Protein–Protein Interaction Network Analysis

Protein–protein interaction network was predicted with STRING (https://string-db.org/, accessed on 29 July 2022) and gene ontologies of the largest subnetwork were analyzed [23]. For the protein–protein interaction network, the confidence (score) cutoff and maximal additional interactions were set as 0.40 and 0, respectively. Data were visualized using Cytoscape (version 3.9.1, Seattle, WA, USA) [24]. The following gene ontology terms were included: cellular component, molecular function, biological processes, KEGG pathways, and REACTOME. Statistical significance was set as *p*-value < 0.05. For visualization, only clusters with more than 4 nodes are shown.

### 2.8. Immunohistochemical Staining

Five µm thick formalin-fixed paraffin-embedded tissue were deparaffinized, rehydrated and incubated in 0.3% (*w*/*v*) H_2_O_2_ in dH_2_O for 30 min to block endogenous peroxidase activity. Heat-induced antigen retrieval was carried out in 10 mM citrate buffer (pH 6.0) or Tris/EDTA buffer (pH 9.0). Tissue was then stained according to standard protocols using the antibodies listed in Table 2. Immunopositivity was detected using 3,3′-diaminobenzidine chromogen or chromogen-bound Alexa Fluor^®^-labelled secondary antibodies. Hematoxylin or DAPI-Fluoromount-G^®^ (ITK diagnostics, Uithoorn, The Netherlands) were used as counterstains. Images were taken using a Leica DM4000B light microscope or a Leica DM5000B fluorescence microscope.

### 2.9. Astrocyte Morphology Analysis

Astrocyte morphology was assessed on GFAP-stained brain tissue using the ImageJ Sholl analysis plug-in. GFAP-positive astrocytes were manually traced. Sholl analysis was then performed, in which concentric rings with increasing sizes were placed around the astrocytes starting from the center of the cell body [26]. The distance between each concentric ring was 3 µm. The following parameters were analyzed: total number of intersections, maximum process length and process thickness.

## 3. Results

### 3.1. Proteomic Map of the VWM Cortex

**Figure 1:**
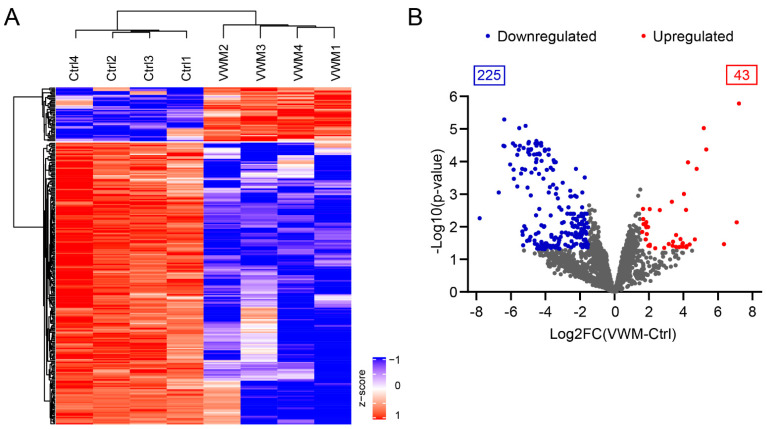
**VWM cortex shows substantial differences from control cortex at the protein level**. (**A**) Heat map of z-scored abundances (LFQ intensities) of the 268 significantly differentially expressed proteins in VWM cortex compared to control cortex. Hierarchical clustering revealed 2 separate expression clusters. (**B**) Differential protein expression in control versus VWM cortex. Volcano plot displaying significant differentially expressed proteins with at least ±1.5-fold change. Proteins downregulated and upregulated in VWM are colored in blue and red, respectively (adj. *p*-value *<* 0.05). Not significantly differentially expressed proteins (adj. *p*-value > 0.05) and proteins with fold changes between ±1.5 are colored in gray.

To explore the proteome of the VWM cortex, high-resolution-mass-spectrometry was employed. We included the pan-cortical tissue of the middle frontal gyrus from 4 VWM subjects and 4 controls (see Table 1). In total, 3239 proteins were identified (Table S1). Of these proteins, 268 were differentially expressed (log 2FC at least ±1.5 and BH adjusted *p*-value < 0.05) in VWM compared to the control (Table S2). The hierarchical clustering analysis of these proteins showed that the VWM samples were clearly separated from the control samples, forming distinct clusters, in which samples from biological replicates aggregated together. In total, 2 protein clusters were identified: cluster 1 contained 43 proteins with high expression in the VWM cortex; and cluster 2 contained 225 proteins with low expression in the VWM cortex (Figure 1 and Table S3).

Considering that our proteomics analysis was performed on the whole cortex of the frontal middle gyrus, the identified proteins may represent changes in multiple cell types. We therefore next examined cell-type-enriched protein expression changes in the cortices of VWM patients. In order to explore this, a transcriptomic dataset from human cortical fetal astrocytes, mature astrocytes, neurons, oligodendrocytes and microglia was analyzed and compared to our proteomic data [22]. Amongst the 43 upregulated proteins, 6 were enriched in fetal astrocytes, and 2 in neurons. Of the 225 downregulated proteins, 4 were enriched in fetal astrocytes, 33 in mature astrocytes, 16 in neurons, 10 in oligodendrocytes and 5 in microglia (Table S4). A Fisher’s exact test revealed that amongst the overexpressed proteins, there was an enrichment in fetal astrocyte-enriched proteins (*p* = 0.039), while amongst the underexpressed proteins, there was an enrichment for mature astrocytes (*p* = 4.5 × 10^−20^), oligodendrocytes (*p* = 2.3 × 10^−4^) and neurons (*p* = 0.017).

### 3.2. Protein–Protein Interaction Network

**Figure 2:**
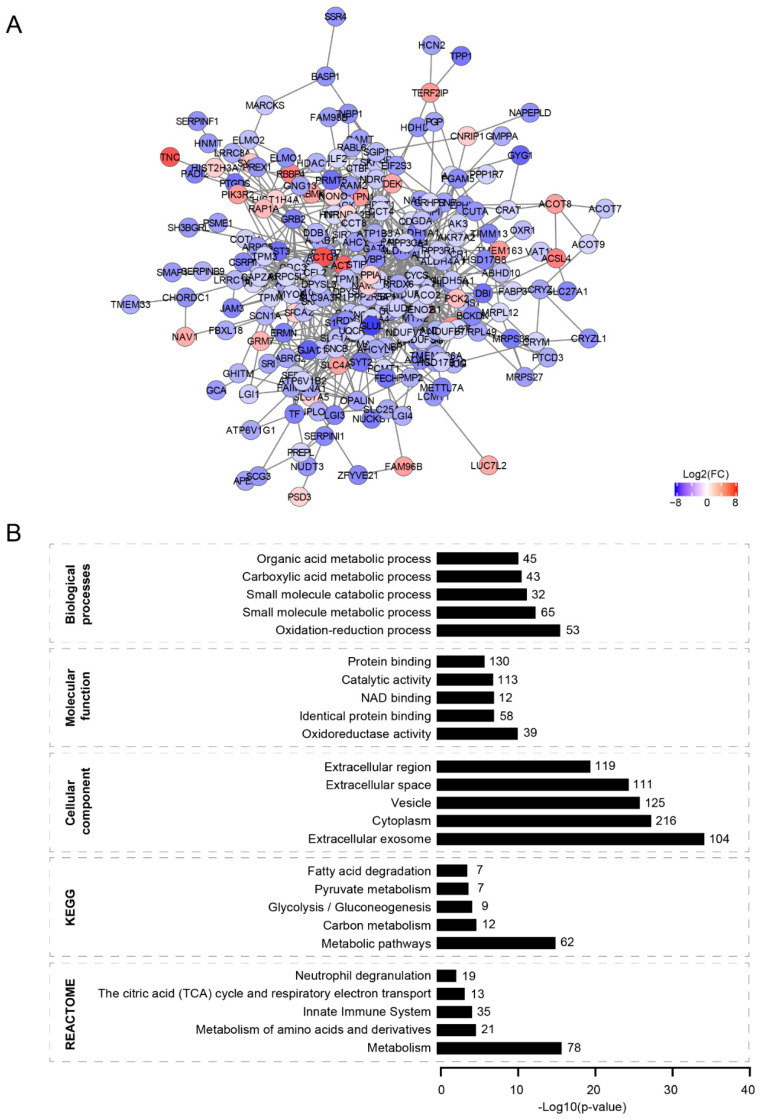
**Protein–protein interaction network of differentially expressed proteins in VWM cortex compared to control cortex.** (**A**) Protein–protein network of differentially expressed proteins in VWM cortex. The network consists of a single major component (largest subnetwork), harboring 234 of the differentially expressed proteins we identified. For visualization, only clusters with more than 4 nodes are shown. Red and blue nodes indicate upregulated and downregulated proteins, respectively. (**B**) Gene ontologies analysis of proteins clustered in the largest subnetwork. Graphs show the top 5 enriched terms (ranked by −log10 (*p*-value)) for each gene ontology. The number of proteins in each term is listed.

To understand the organization of the differentially expressed proteins, we applied a protein–protein interaction network approach. This uncovered a protein network with one major connected component harboring 234 proteins, in which over- and under-expressed proteins clustered together (Figure 2A). We also observed several unconnected nodes, accounting for 34 proteins (Table S5A). Next, to gain insight into possible biological functions and pathways altered in the VWM cortex, a gene ontology enrichment analysis was performed on the differentially expressed proteins (Table S5B). In VWM cortices, differentially expressed proteins were mainly located in the extracellular exosomes, cytoplasm, vesicles and extracellular space (Figure 2B). Some of the network proteins were also enriched for gene ontology terms related to cellular metabolism, such as the oxidation–reduction process, oxidoreductase activity and NAD binding (Figure 2B). Consistently, we found an overrepresentation of terms for metabolic pathways, particularly glycolysis/gluconeogenesis, pyruvate metabolism, fatty acid degradation, the metabolism of amino acids and derivatives, the citric acid (TCA) cycle and respiratory electron transport body (Figure 2B).

**Figure 3:**
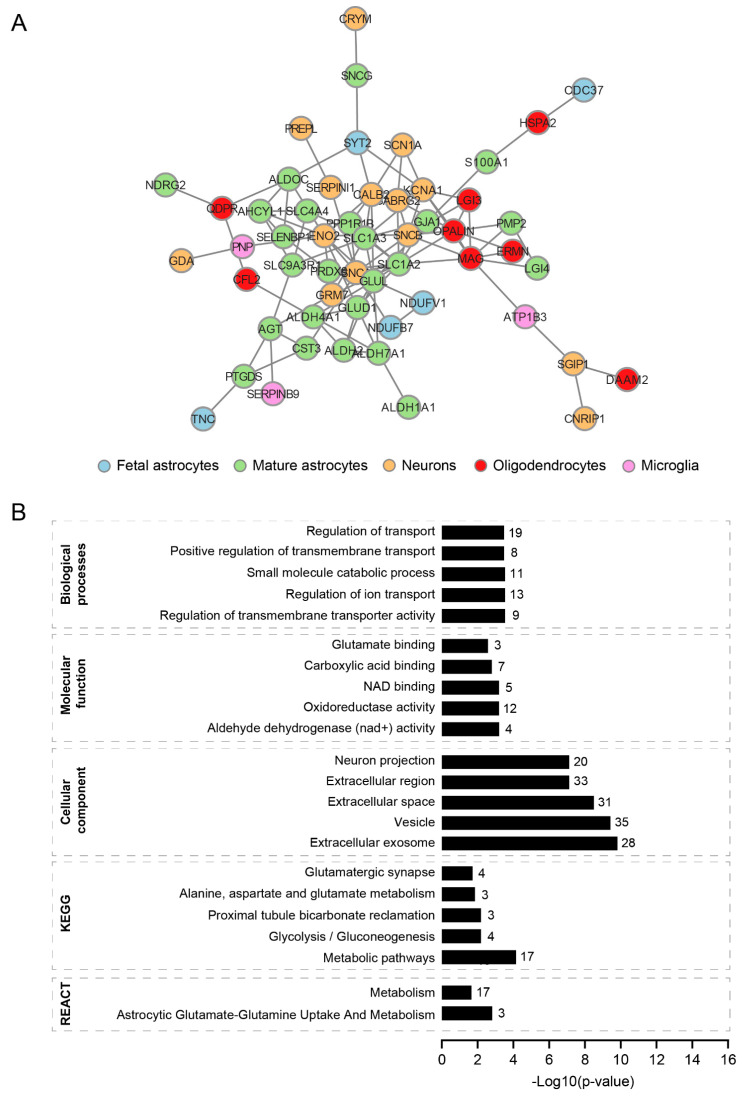
**Protein–protein interaction network of differentially expressed proteins in different cell types.** (**A**) Protein–protein network of differentially expressed and cell-type-enriched proteins in VWM cortex. The network consists of a large subnetwork, harboring 54 cell-type-enriched proteins. For visualization, only clusters with more than 4 nodes are shown. (**B**) Gene ontologies analysis of proteins clustered in the largest subnetwork. Graphs show the top 5 enriched terms (ranked by −log10 (*p*-value) for each gene ontology. The number of proteins in each term is listed. In (**B**), REACT is an abbreviation for REACTOME.

The cell type enrichment analysis of significantly differentially expressed proteins revealed a distinct cell type involvement in VWM cortices (Table S4). To further stratify the organization of the differentially expressed proteins from mixed cell populations, we repeated the analysis, only taking into account the proteins that we identified as enriched in different cell types. Among the 76 brain cell-type-enriched proteins, we found that 54 clustered together, forming a subnetwork (Figure 3A and Table S6A). A subsequent gene ontologies analysis revealed that these cell-type-enriched proteins were located in extracellular exosomes, vesicles, extracellular space, extracellular regions and neuron projections (Figure 3B and Table S6B). The protein network also showed enrichment for biological processes related to transport (Figure 3B and Table S6B). Notably, some proteins in this network were also enriched for gene ontology terms associated with cellular metabolism, especially oxidoreductase activity, with 12 out of 54 cell-type-enriched proteins overrepresented in that term. Amongst these 12 proteins, the majority were astrocyte-enriched, suggesting that cellular metabolism might be particularly affected in these cells in the VWM cortex (Table S6B). In summary, our findings suggest that the majority of the differentially expressed proteins physically or functionally interact with each other (Figure 2 and Figure 3). The protein–protein interaction network analysis only revealed a single large subnetwork and no other specific clusters were evident.

### 3.3. Localization and Distribution of Differentially Expressed Proteins in VWM

To confirm our proteomic findings, we selected the top 3 up- and downregulated proteins in VWM compared to the control cortex (Table S2). The top 3 upregulated proteins were actin gamma 1 (ACTG1), tenascin C (TNC) and actin beta (ACTB). The top 3 downregulated proteins were glutamate-ammonia ligase (GLUL), heat-responsive protein 12 (HRSP12) and gap-junction alpha-1 protein (GJA1, also known as connexin-43). All proteins except for HRSP12 were part of the largest subnetwork (Figure 2A). Notably, based on the cell type enrichment analysis (Table S4), some proteins were enriched for fetal astrocytes (i.e., TNC) and mature astrocytes (i.e., GLUL, HRSP12 and GJA1). Neither ACTG1 and ACTB were assigned to a particular cell type (Table S4). To gain insight into the localization and distribution of these proteins in the cortex of VWM patients and the controls, immunohistochemistry was performed on all VWM patients and the controls (the results were comparable in each group).

**Figure 4:**
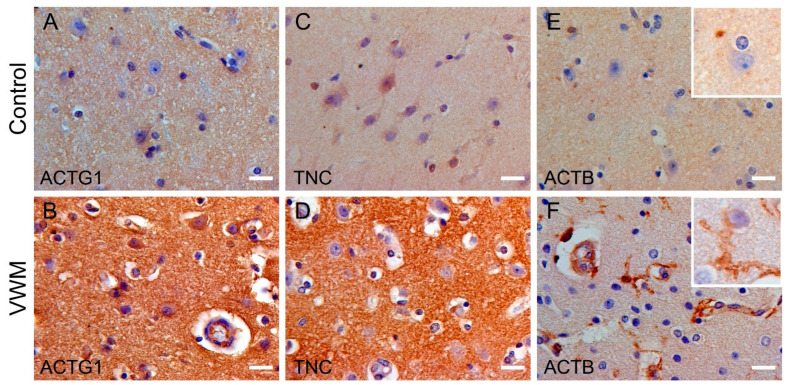
**Expression pattern of ACTG1, TNC and ACTB in control and VWM cortices.** (**A**–**F**) Immunohistochemistry against ACTG1, TNC and ACTB in control cortex (**upper panel**) and VWM cortex (**bottom panel**). Insets in (**E**,**F**) are higher magnification images, showing cellular immunoreactivity of ACTB in VWM cortex, but not in control cortex. Blue stain indicates nuclei. Scale bars: 20 µm. Depicted images are stains of cortical tissue of control 2 and VWM 1.

We started with the top 3 upregulated proteins: ACTG1, TNC and ACTB. The immunohistochemistry against ACTG1 showed low expression in the control cortex and high expression in the VWM cortex. ACTG1 expression was found in between cell bodies, but also in neurons and in blood vessels (Figure 4A,B). TNC showed increased immunoreactivity in the cortex of VWM patients. In the control cortex, TNC expression was detected in only a few cells. Based on nuclear size and morphology, these are presumably glia and neurons. In the VWM cortex, TNC expression was predominantly extracellular (Figure 4C,D). Low immunoreactivity for ACTB was present in the control cortex. In the VWM cortex, ACTB immunoreactivity was increased, consistent with the differences observed in our proteomics findings. Remarkably, the expression was restricted to non-neuronal cells, possibly glia, based on morphology (Figure 4E,F).

**Figure 5:**
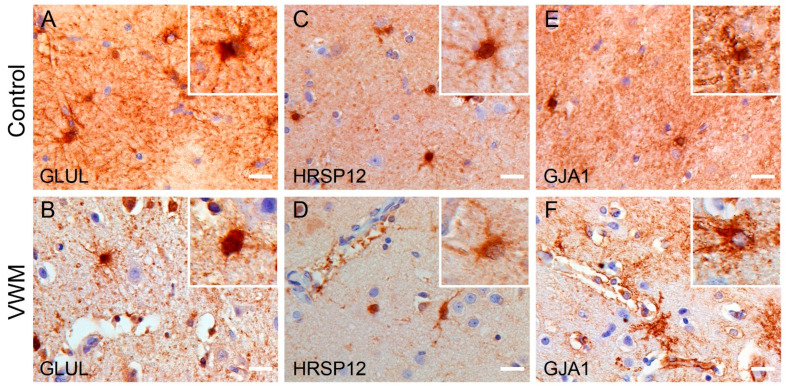
**Expression pattern of GLUL, HRSP12 and GJA1 in control and VWM cortices.** (**A**–**F**) Immunohistochemistry against GLUL, HRSP12 and GJA1 in control cortex (**upper panel**) and VWM cortex (**bottom panel**). Insets are higher magnification images, showing immunoreactive cells with astrocyte-like morphology. Blue stain indicates nuclei. Scale bars: 20 µm. Depicted images are stains of cortical tissue of control 4 and VWM 3.

Next, we looked into the expression patterns of the top 3 downregulated proteins in the VWM cortex: GLUL, HRSP12 and GJA1. GLUL was localized in astroglia-like cells in both the control and VWM cortices. The immunoreactivity in between cells, however, was decreased in the VWM cortex compared to the control cortex. Notably, in the control cortex, GLUL-positive cells were extensively branched, whereas in the VWM cortex, they were less complex in morphology (Figure 5A,B). HRSP12 expression was found in astrocyte-like cells in the control and VWM cortices. Overall immunoreactivity appeared less in the VWM cortex compared to the controls (Figure 5C,D). GJA1 was detected in cells with astrocyte morphology in both the control and VWM cortices. The immunoreactivity, however, was decreased in between cells in the VWM cortex. Notably, GJA1 and GLUL share similar expression patterns. In this, GJA1-positive cellular processes also appeared more complex in morphology in the control cortex compared to those in VWM (Figure 5E,F).

**Figure 6:**
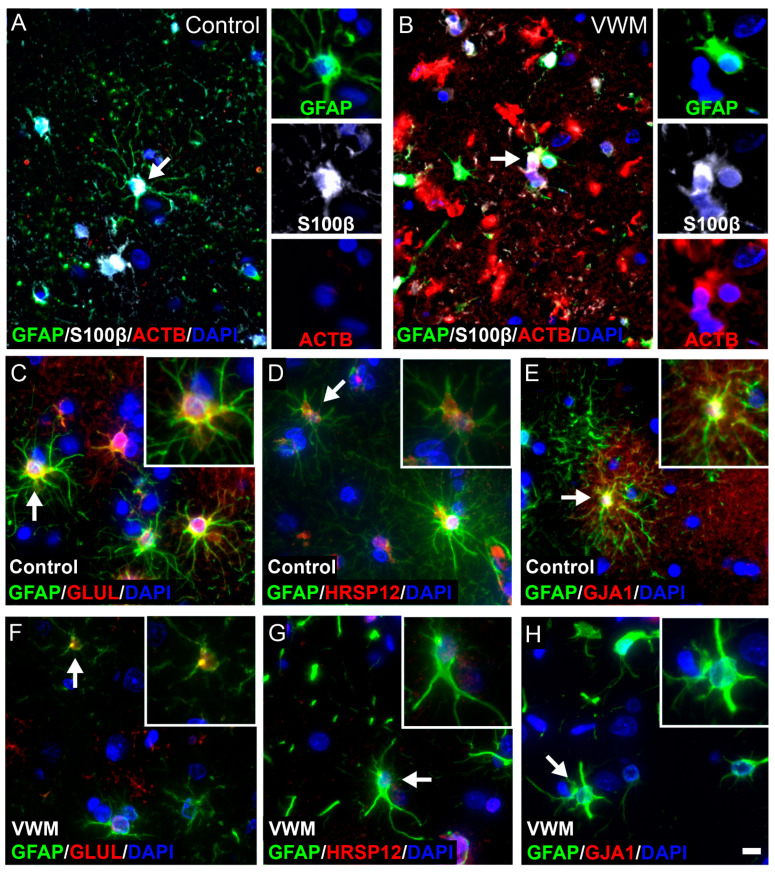
**Cellular distribution of ACTB, GLUL, HRSP12 and GJA1 in control and VWM cortices.** Triple stain against GFAP (green) and S100β (white) with ACTB (red) in control (**A**) and VWM (**B**) cortices. Double stain against GFAP (green) with GLUL, HRSP12 and GJA1 (red) in control (**C**–**E**) and VWM (**F**–**H**) cortices. Cells indicated by white arrows are displayed in insets. Blue stain indicates nuclei. Scale bar: 10 µm. Depicted images are stains of control 4 and VWM 2.

Notably, amongst the 6 validated proteins, 4 were found expressed in cells with glia morphology, particularly cells with astrocytic morphology and nuclear features. These proteins include ACTB, GLUL, HRSP12 and GJA1. We next set out to confirm their expression in astrocytes. Double-labeling with GFAP confirmed the expression of GLUL, HRSP12 and GJA1 in astrocytes in the control cortex (Figure 6), whereas ACTB was absent, consistent with the immunohistochemistry (Figure 4E). In VWM, the expression in astrocytes was only partly confirmed (Figure 6). The ACTB expression was clearly upregulated in the VWM cortex, with the expression restricted to cells (Figure 6B). These cells were, however, not positive for GFAP. Since cortical astrocytes might express GFAP below the level of antibody detection, we also stained for Ca^2+^- and Zn^2+^-binding protein S100β [27]. Double-labeling with S100β showed little co-expression with ACTB, indicating that ACTB is expressed mainly in other cells than astrocytes (Figure 6B). GLUL-positive cells co-localized with GFAP in VWM (Figure 6F), whereas HRSP12 and GJA1 expression was not detected (Figure 6G,H). Since HRSP12 and GJA1 were downregulated in the VWM cortex, the absence of these proteins might be explained by an antibody level below the detection threshold for immunofluorescence.

### 3.4. Dysmorphic and Immature Astrocytes in the VWM Cortex

Thus far, some of the proteins with the most prominent changes in expression in the VWM cortex were localized in astrocytes, especially GLUL, HRSP12 and GJA1 (Figure 5 and Figure 6). This, combined with the central role of astrocytes in VWM white matter pathology, prompted us to focus on this cell type in the cortex.

**Figure 7:**
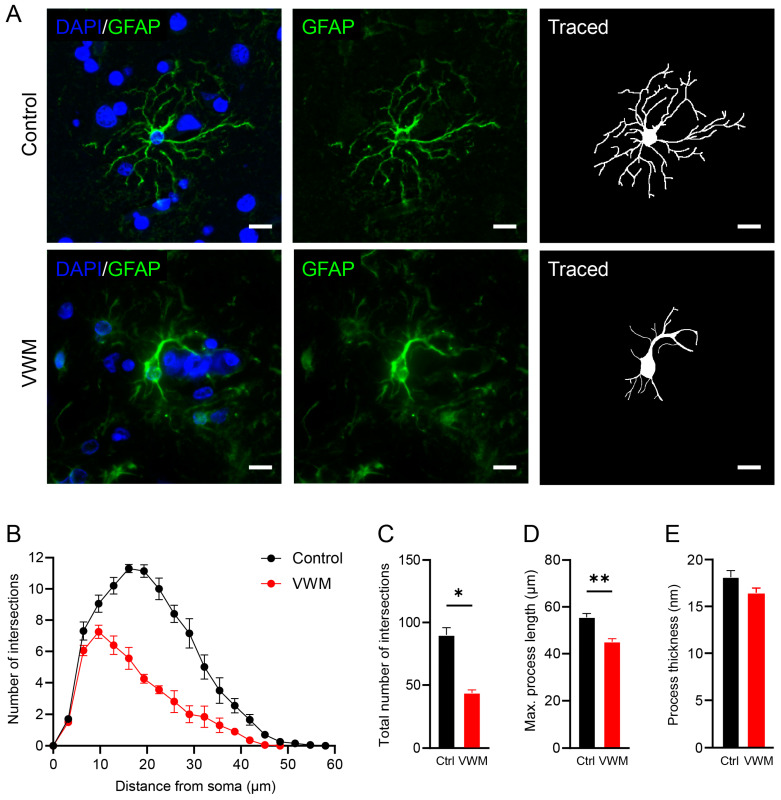
**Astrocytes in the VWM cortex show reduced morphological complexity.** (**A**) Immunostaining of control and VWM cortices showing GFAP-positive astrocytes (green) and their respective tracings. Blue stain indicates nuclei. Scale bars: 10 µm. Depicted images are stains of cortical tissue of control 1 and VWM 1. (**B**) Sholl diagram of astrocytes in the cortex of control and VWM brains. Diagram shows number of processes intersecting with concentric rings per 3 µm, starting from the cell body. (**C**) Total number of intersections. (**D**) Maximum process length. (**E**) Process thickness. * *p*-value *<* 0.05, ** *p*-value *<* 0.01.

To investigate whether cortical astrocytes in VWM show abnormalities, we performed a morphological analysis [26]. Typically, astrocytes in the cortex are highly branched with bushy processes [28], consistent with our control findings (Figure 7A). In the control, cortical astrocytes were only visible in layer I close to the glia limitans and around blood vessels. In the VWM cortex, astrocytes were detectable in all layers and were much less complex in morphology (data not shown). Although not as severely affected as the astrocytes in the VWM white matter (Figure S1), cortical astrocytes showed significantly less branched and shorter processes than those in controls (Figure 7B–D). No changes in process thickness were observed (Figure 7E).

**Figure 8:**
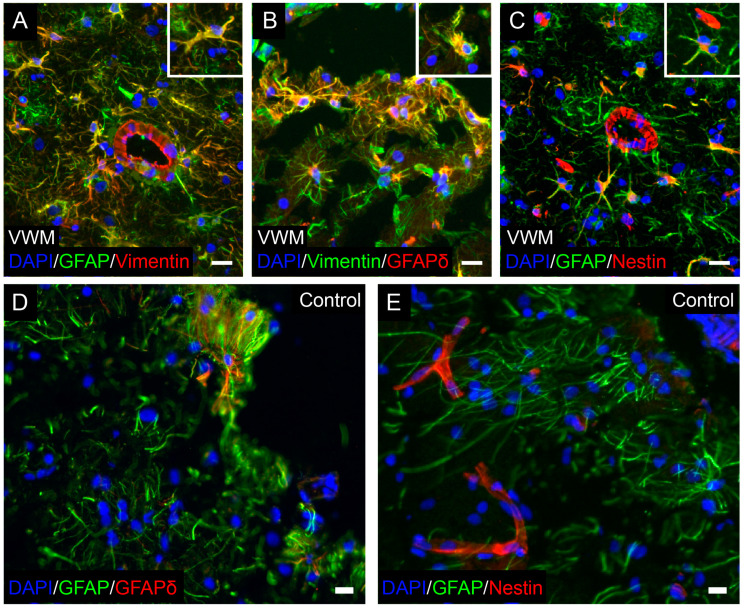
**Astrocytes in the VWM cortex are immature and have a disrupted intermediate filament network.** Double stain in VWM cortex against (**A**) GFAP (green) and vimentin (red), (**B**) vimentin (green) and GFAPδ (red), and (**C**) GFAP (green) and nestin (red). Insets show double-labeled cells. Double stain in control cortex for (**D**) GFAP (green) and GFAPδ (red), and (**E**) GFAP (green) and nestin (red). Blue stain indicates nuclei. Scale bars: 20 µm. Depicted images are stains of control 4 and VWM 3.

Specifically in VWM, and as in white matter, no reactive gliosis was seen in the cortex. VWM cortical astrocytes also co-expressed the intermediate filament protein vimentin (Figure 8A). Interestingly, VWM astrocytes throughout the cortex were also strongly immunopositive for GFAPδ, indicating a disrupted intermediate filament network (Figure 8B). Furthermore, they expressed the intermediate filament protein nestin, suggesting immaturity (Figure 8C). Very low, if any, GFAPδ and nestin expressions were found in the control cortical astrocytes (Figure 8D,E).

## 4. Discussion

In this study, we provide a comprehensive analysis of the VWM cortex. Our data show that the cortex, rather than being spared, is affected in VWM. The analysis of the cortices in the VWM patients at the protein level revealed substantial differences compared to those in the control subjects. Numerous differentially expressed proteins formed a large protein–protein interaction network. We also show that differentially expressed proteins enriched in different cell types were linked to each other. These findings indicate that the majority of differentially expressed proteins identified in our proteomics study physically or functionally interact with each other. The subsequent gene ontology analysis identified enrichment for various terms, including those associated with cellular metabolism. Our findings also hint at the likely astrocytic involvement in the VWM cortex. Some of the proteins with the most prominent changes were found in astrocytes. Additionally, a further analysis showed that VWM cortical astrocytes are immature and exhibit morphological changes resulting in a less complex structure.

To better understand cortical pathology in VWM, we first mapped the protein expression patterns using high-resolution-mass-spectrometry-based proteomics. We identified 268 proteins associated with VWM. The cell type enrichment analysis revealed that some of the higher-expressed proteins in the VWM cortex were most enriched in fetal astrocytes. The set of lower-expressed proteins in the VWM cortex were enriched in mature astrocytes and, to a lesser extent, also oligodendrocytes and neurons. These findings suggest that multiple cell types contribute to or are affected in the VWM cortex. Moreover, it may also reflect changes in primary and secondary (e.g., cell autonomous and non-cell autonomous) cellular pathology. Notably, the enrichments found in the VWM cortex are consistent with the known pathology driving brain white matter degeneration of the disease, with astrocytes having a primary role and secondary effects on both oligodendrocytes and axons (neurons) [29]. This suggest that a similar cellular pathology could be present in the cortex.

Through the use of a protein–protein interaction network and a subsequent gene ontology term-enrichment analysis, we identified biological functions potentially altered in VWM cortices. In particular, there was enrichment in terms that were suggestive of altered cellular metabolism, particularly mitochondrial functions. Mitochondrial effects in astrocytes and oligodendrocytes have been described in experimental VWM mouse models [30,31,32,33]. This correlates with both glial cell types being predominantly affected in VWM [3,11]. We now add data suggesting that the mitochondrial function is changed in the cortices of VWM patients, hinting at a potential general defect in the VWM brain. Remarkably, an imaging phenocopy of VWM is mitochondrial leukodystrophy, due to respiratory chain complex II deficiency and *SDHA* mutations [34,35]. Both diseases feature diffuse white matter signal changes with cavitations in the cerebral white matter. It could be argued that a primary functional impairment of astrocytes is involved in both disorders, halting glia scarring. Currently, we do not know if mitochondrial changes are linked with the deregulated ISR in VWM gray matter astrocytes [10]; the ISR has been implicated in regulating several metabolic pathways [36].

Amongst the identified differentially expressed proteins, 6 proteins with the most prominent changes in expression were selected and validated by immunohistochemistry. Interestingly, the expression pattern of some of these proteins appear to coincide with that of astrocytes, in particular the lower-expressed proteins GLUL, HRSP12 and GJA1. GLUL and GJA1 are astrocytic proteins. GLUL is an enzyme involved in the conversion of glutamate and ammonium to glutamine, also known as glutamate metabolism. Glutamine plays an important role in cell metabolism, particularly in the tricarboxylic acid cycle and the biosynthesis of several amino acids, nucleotides and glutathione [37,38,39,40]. GJA1 (also known as connexin-43) is a transmembrane protein involved in the coupling of astrocytes to form domains within the so-called pan-glial syncytium [41,42,43]. Gap junctions allow the trafficking of molecules, including ions and metabolites, between cells [41,42,43]. HRSP12, on the other hand, is a reactive intermediate deaminase A protein that has not been associated with astrocytes before. Its functions are still unknown, but it is proposed to participate in cellular metabolism, such as 2-iminobutanoate deaminase activity and mRNA catabolic processes [44,45]. It should be noted that amongst the validated proteins, GLUL and HRSP12 relate to metabolic processes, supporting the possibility of altered cell metabolism. Other validated proteins (i.e., ACTG1 and TNC) were found in the extracellular matrix.

The fact that some of the most prominent changes were specifically observed in astrocytes suggests that cortical involvement in VWM may also be driven by changes in this cell type. The presence of dysmorphic astrocytes is a typical feature of VWM [3,11,15]. Astrocytes residing in the affected white matter usually have short blunt processes compared to the fine, long processes seen in the controls [3,11]. They remain immature and typically fail in mature astrocytic functions, including reactive gliosis and glial scarring [11]. Their intermediate filament composition is altered, with an absolute overexpression of GFAPδ, which could explain their dysmorphic appearance [11]. We now add to these findings and show that, although to a lesser degree than in the white matter, VWM cortical astrocytes are affected as well. In this, VWM cortical astrocytes exhibit abnormal morphology with less branched and shorter cellular processes. These astrocytes also appear immature, with an overexpression of GFAPδ, nestin and vimentin. The concurrent downregulation of nestin and vimentin is associated with astrocytic differentiation [46,47,48], supporting the possibility of an altered astrocytic maturation in VWM. Additionally, the cell type enrichment analysis showed that amongst the higher-expressed proteins in VWM, 6 were enriched in fetal astrocytes. Furthermore, amongst the lower-expressed proteins, 4 were enriched in fetal astrocytes and 33 were enriched in mature astrocytes. Maturation is a dynamic process, regulated by increasing the expression of some proteins and decreasing the expression of other proteins [49]. There is also no hint of reactive gliosis in the VWM cortex. The hallmarks of reactive gliosis are the induction of nestin and upregulation of GFAP and vimentin [49]. We found that VWM cortical astrocytes, such as reactive astrocytes, robustly express nestin and vimentin, but do not upregulate the *total* GFAP [11]. Additionally, in reactive astrocytes, GFAPδ represents only a small fraction of the total GFAP, not always immunohistochemically detectable, whereas GFAPδ expression is clearly visible in VWM cortical astrocytes. Moreover, VWM astrocytes are not hypertrophic. They do not show an increase in number nor in length of cellular processes, but rather the opposite [49]. All together, these data suggest immaturity rather than reactive gliosis. Our findings therefore suggest a potential role of astrocytes in both gray and white matter pathology in VWM. In line with this, a previous study using human-induced pluripotent stem cells further demonstrated astrocytic involvement in VWM, with white matter astrocytes showing higher vulnerability than gray matter astrocytes [50]. It remains unclear whether cortical astrocyte pathology relates to white matter pathology in VWM. In VWM, white matter astrocytes are severely abnormal. Nonetheless, the cortex has always reported to be normal [29]. Our new data show that gray matter astrocytes are also affected, although to a lesser degree. Astrocytes in the cortex, however, are completely different from white matter astrocytes [51]. The possibility of a knock-on effect cannot be excluded and warrants additional studies.

Some limitations apply to this work. Although VWM is one of the more prevalent leukodystrophies [1], it is still a very rare disease [2]. This results in the limited availability of human brain tissue. Our sample size was therefore restricted to 4 VWM cases. However, it should be noted that all patients included in this study had a childhood-onset form of VWM with comparable pathologies characterized by a lack of myelin, meager reactive gliosis and no glial scarring [3,4,5,11]. Some patients survive longer, while others die younger. The difference is in part explained by external factors (e.g., infections). Since there is also a limited availability of control human brain tissue of a high quality suitable for proteomics, especially from young healthy subjects, our sample size was restricted to 4 control cases. Notably, such tissue is extremely rare, especially that obtained within a short post-mortem period.

Another potential concern is the mismatch in ages between the patient and control groups with only 1 patient being of similar age. It is therefore possible that the changes in the abundance of certain proteins may be in part caused by aging. The substantial differences between the controls and the 29-year-old VWM patient and the fact that all patients cluster together, however, suggest that aging does not significantly impact the changes found.

The distribution and localization of several proteins identified by proteomics were mapped by immunohistochemistry. A validation by immunoblotting was not done because of several reasons. Firstly, the proteomics data indicate the relative levels of protein expression. Secondly, the tissue available was limited. Thirdly, the immunoblotting does not elucidate the regional expression. Although immunohistochemistry is not absolutely quantitative, it gives insight into the cell types expressing the proteins, and the location within the cortex these proteins are expressed.

The expression patterns of proteins selected for validation did not always coincide in tissue sections developed with diaminobenzidine chromogen or fluorochrome. In particular, strong cellular immunoreactivity for some proteins was detected with chromogen detection, whereas we were not able to confirm their expression with immunofluorescence. This might be due to the difference in the antibody detection method and threshold.

Another limitation is that our proteome analysis was performed on the whole tissue, and the data may represent changes in multiple cell types. To study the involvement of brain cell types in VWM, we applied a cell type enrichment analysis. This analysis is typically used on bulk transcriptomic datasets. There are currently no datasets available of single-cell-type proteomics of the human brain. For this reason, we compared our proteome dataset with a published human mRNA sequencing dataset [22]. It is, however, well known that the correlation between mRNA and protein levels is not straightforward, resulting in differences in specificity across cell types at the protein and mRNA levels [52,53]. The results should therefore be interpreted with caution. Future single-cell-type proteomics studies may further help us confirm our results.

## 5. Conclusions

In conclusion, our data support the notion that the frontal cortex is also affected in VWM. This has to be taken into account when developing therapeutic strategies.

## Figures and Tables

**Table 1:** Demographic features of controls and patients.

Subject	NBB Reference No.	Age at Death	Molecular Diagnosis
Control 1	2003–085	24 years	-
Control 2	2017–029	23 years	-
Control 3	2018–010	21 years	-
Control 4	2018–066	35 years	-
VWM 1	-	29 years	ε, Thr91Ala/Thr91Ala *
VWM 2	-	10 years	ε, Arg113His/Ala403Val *
VWM 3	-	6 years	ε, Thr91Ala/Val437Met *
VWM 4	-	12 years	ε, Thr91Ala/Ala403Val *

* Mutation: mutant eIF2B subunit and amino acid changes are indicated.

**Table 2:** List of antibodies used for immunohistochemistry.

Antibody	Dilution	Vendor	Catalogue No.
GFAP	1:1000	DAKO	Z0334
GFAP	1:750	Merck Millipore	AB5541
Vimentin	1:2000	Clone V9 *	-
GFAPδ	1:500	†	-
Nestin	1:200	Millipore	MAB5623
GJA1	1:100	Sigma-Aldrich	C6219
GLUL	1:5000	ThermoFisher	PA5-81505
HRSP12	1:100	ThermoFisher	PA5-54622
ACTG1	1:100	Abcam	ab123034
TNC	1:200	Abcam	ab108930
ACTB	1:1500	Abcam	ab8226
S100β	1:100	DAKO	Z311

* Manufactured at the department of Pathology, Amsterdam UMC, The Netherlands. † Manufactured at the Netherlands Institute for Neuroscience, Amsterdam, The Netherlands [25].

## Data Availability

The mass spectrometry proteomics data have been deposited into the ProteomeXchange Consortium via the PRIDE [54] partner repository with the dataset identifier PXD030831. Other datasets used during this study are available from the corresponding author on reasonable request.

## Supplementary Materials

The following supporting information can be downloaded at: https://www.mdpi.com/article/10.3390/cells11223581/s1, Figure S1: Astrocytes show region-dependent vulnerability in VWM gray and white matter brain tissue; Table S1: List of all detected proteins in control and VWM cortex; Table S2: List of differentially expressed proteins in control versus VWM cortex; Table S3: Results obtained from hierarchical clustering analysis of significantly differentially expressed proteins; Table S4: List of cell-type-enriched differentially expressed proteins in VWM cortex; Table S5: Protein–protein interaction network analysis of significantly differentially expressed proteins; Table S6: Protein–protein interaction network analysis of significantly differentially expressed proteins in different cell types.
