# Supplementary material for: Cortical Pathology in Vanishing White Matter
Source: Cells. 2022 Nov 12;11(22):3581. doi: 10.3390/cells11223581 (PMC9688115; doi:10.3390/cells11223581)
Supplement: Supplementary file 1 [file cells-11-03581-s001.zip › cells-1858119-Figure S1.pdf]

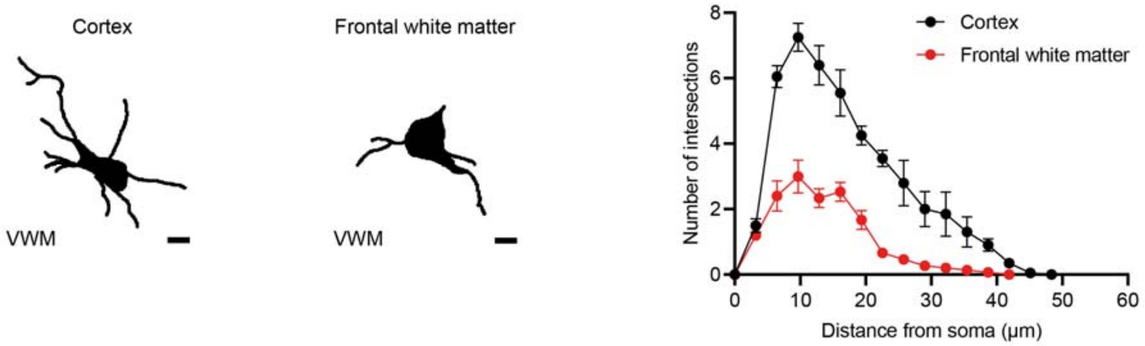

Figure S1. Astrocytes show region-dependent vulnerability in VWM gray and white matter brain tissue.
